# Impairments that Influence Physical Function among Survivors of Childhood Cancer

**DOI:** 10.3390/children2010001

**Published:** 2015-01-08

**Authors:** Carmen L. Wilson, Prasad L. Gawade, Kirsten K. Ness

**Affiliations:** Department of Epidemiology and Cancer Control, St. Jude Children’s Research Hospital, 262 Danny Thomas Place, MS-735 Memphis, TN 38105, USA; E-Mails: pgawade@amgen.com (P.L.G.); kiri.ness@stjude.org (K.K.N.)

**Keywords:** childhood cancer, cancer survivor, late effects, BMI, BMD, musculoskeletal, cardiopulmonary, vision, physical function, neuromotor

## Abstract

Children treated for cancer are at increased risk of developing chronic health conditions, some of which may manifest during or soon after treatment while others emerge many years after therapy. These health problems may limit physical performance and functional capacity, interfering with participation in work, social, and recreational activities. In this review, we discuss treatment-induced impairments in the endocrine, musculoskeletal, neurological, and cardiopulmonary systems and their influence on mobility and physical function. We found that cranial radiation at a young age was associated with a broad range of chronic conditions including obesity, short stature, low bone mineral density and neuromotor impairments. Anthracyclines and chest radiation are associated with both short and long-term cardiotoxicity. Although numerous chronic conditions are documented among individuals treated for childhood cancer, the impact of these conditions on mobility and function are not well characterized, with most studies limited to survivors of acute lymphoblastic leukemia and brain tumors. Moving forward, further research assessing the impact of chronic conditions on participation in work and social activities is required. Moreover, interventions to prevent or ameliorate the loss of physical function among children treated for cancer are likely to become an important area of survivorship research.

## 1. Introduction

There are nearly 16,000 new cases of childhood and adolescent cancer diagnosed in the United States each year [1]. Fortunately, improvements in treatment, including multimodal therapy and hospital care, have improved survival such that over 80% of children diagnosed with a malignancy will survive at least five years [2]. However, childhood cancer survivors are at increased risk of developing chronic health conditions, some of which manifest during or soon after treatment whereas others emerge years after therapy [3,4,5]. Recognizing chronic health conditions among childhood cancer survivors has received considerable attention in the past decade and has led to the development of exposure-based medical screening guidelines [6], and, in some instances, the modification or elimination of treatments that cause severe toxicity [7]. Nevertheless, among the approximately 420,000 childhood cancer survivors alive in the United States today [8], 80.5% experience a severe, life-threatening, or disabling chronic condition by age 45 years [3]. The presence of a chronic health condition increases risk for functional impairments by 3.25 (95% confidence interval [CI]: 2.97–3.55) and for activity limitations by 3.20 (95% CI: 2.91–3.53) [9].

Acute toxicities develop during cancer therapy and are usually related to the immediate effects of cancer, chemotherapy, and radiotherapy on rapidly dividing cells. Most children recover from these acute problems [10,11] such as neutropenia, nausea, vomiting, and growth deceleration. However, clinically undetectable organ system damage may subtly reduce physiologic reserve, such that the toxicity does not appear until growth and maturity require increasing capacity. Acute toxicities may also promote the development and maintenance of an unhealthy lifestyle among children with cancer. Prolonged hospitalization, bed rest, activity restrictions, and weight gain associated with poor nutrition during treatment may promote sedentary behavior and poor food choices, compounding existing organ system damage and impeding an active lifestyle. Because many chronic conditions described among childhood cancer survivors are less common in members of the non-cancer population who adopt a healthy lifestyle [12,13], promoting healthy behaviors among childhood cancer survivors is important. To do this, an understanding of organ-system toxicities that affect physical performance and function among childhood cancer survivors is essential, as preventive and rehabilitative measures need to accommodate the unique needs of survivors so they can fully participate in behaviors that promote their health and well-being.

In this review, we discuss treatment-related organ impairments in the endocrine, musculoskeletal, neurological, and cardiopulmonary systems and their influence on the mobility and function necessary to participate fully in work, school and community environments.

## 2. Endocrine Complications

### 2.1. Body Composition

Changes in body composition, including increased body mass index (BMI) and percent body fat, are well documented among survivors of acute lymphoblastic leukemia (ALL) and brain tumors [14,15,16,17]. Corticosteroids are associated with increased BMI during and shortly after treatment in children with ALL [18,19,20,21], but whether these changes in body composition persist long term is unknown [15,22,23,24]. Among survivors of childhood cancer, females [16,17,25,26,27,28], those treated with cranial irradiation [29,30], those who have tumors near the hypothalamus or pituitary [31,32], or those who were treated at a younger age [16,33] are at the greatest risk of obesity (see Table 1). Low levels of physical activity [34], reduced energy expenditure during exercise [35], and poor nutrition [36] can also contribute to obesity. Compared with population-based norms, survivors of Hodgkin lymphoma and neuroblastoma, as well as male survivors of non-Hodgkin lymphoma, Wilms tumor, and soft tissue sarcoma, are more likely to have low BMI [37]. Exposure to alkylating agents [37], anthracyclines [37,38], abdominal radiation [39] and hematopoietic stem cell transplant (HSCT) [38,40,41] are associated with low BMI. Low BMI among survivors is thought to be a consequence of low lean body mass rather than low fat mass [23,42,43]. It is suggested that waist circumference or BMI may be inaccurate measures of obesity for individuals previously exposed to radiation involving the abdomen [44].

**Table 1:** Acute and late effects among survivors of childhood cancer.

System	Condition	Risk Factors
Endocrine	Obesity	Cranial irradiation, high caloric intake, low levels of physical activity, female sex
	Short stature	Cranial, spinal, or total body irradiation, young age at treatment
Musculoskeletal	Reduced bone mineral density	Methotrexate, dexamethasone, prednisone, HSCT, growth hormone deficiency, estrogen deficiency, testosterone deficiency, reduced physical activity, low calcium or vitamin D intake, treatment during adolescence
	Deformity (including scoliosis, kyphosis, limb-length discrepancy)	Surgery, spinal, flank, or whole-abdomen irradiation, younger age at treatment
	Osteonecrosis	Dexamethasone, prednisone, HSCT, older age at treatment
Neurosensory	Cataract	Busulfan, dexamethasone, prednisone, eye irradiation
	Other ocular toxicities (e.g., keratitis, telangiectasia, retinopathy, optic chiasm neuropathy, enophthalmos, maculopathy, papillopathy, glaucoma)	Orbital irradiation
	Vertigo	Carboplatin, cisplatin
	Peripheral sensory neuropathy	Carboplatin, cisplatin
Neuromotor	Peripheral motor neuropathy (including areflexia, weakness, foot drop, paresthesia)	Vinblastine, vincristine
	Motor deficits (e.g., hemiparesis paralysis, ataxia, dysarthria, dysphagia, paralysis, seizures)	Cranial irradiation, neurosurgery
Cardiovascular	Cardiomyopathy	Anthracyclines (daunorubicin, doxorubicin, epirubicin, idarubicin, mitoxantrone), chest radiation, female sex, younger age at treatment
	Arrhythmias	Anthracyclines (daunorubicin, doxorubicin, epirubicin, idarubicin, mitoxantrone), chest irradiation
	Atherosclerotic heart disease, congestive heart failure, hypertension, pericarditis, pericardial fibrosis, valvular disease, myocardial infarction	Chest irradiation
	Carotid artery disease	Chest, cervical, or cranial irradiation
	Dyslipidemia	Carboplatin, cisplatin
Pulmonary	Acute respiratory distress	Bleomycin
	Interstitial pneumonitis	Bleomycin, chest irradiation
	Fibrosis	Busulfan, BCNU, CCNU, bleomycin, chest radiation
	Restrictive lung disease, obstructive lung disease	Chest radiation
	Bronchial obliterans, bronchiectasis, chronic bronchitis	HSCT with history of chronic GVHD
	Pulmonary dysfunction	Lobectomy, wedge resection
	Chronic sinusitis	Orbital or nasopharyngeal radiation

**Abbreviations:** BCNU, carmustine; CCNU, lomustine; GVHD, graft-versus-host disease; HSCT, hematopoietic stem cell transplant.

Obesity is an important concern in childhood cancer survivors, as it is associated with increased risk for chronic disease [45] and reduced physical function [46,47,48] in the general population. Furthermore, longitudinal studies of childhood cancer survivors [49] indicate that obese individuals are more likely to become less active over time. A high risk of obesity combined with low levels of physical activity may compound the risk of chronic illness and accompanying physical limitations in survivors of childhood cancer. Although several studies have explored associations between obesity and the risk of metabolic syndrome [30] and cardiovascular health among cancer survivors, few studies have explored the impact of obesity on physical function.

### 2.2. Height

Children with cancer are at risk of poor growth and reduced final height [50,51,52]. Reductions in height standard deviation scores (SDS) have been reported for children receiving therapy for ALL, especially during the first 6–12 months of therapy, and are generally attributed to the effects of chemotherapy and glucocorticoids [53,54,55,56]. Although catch-up growth is observed in the first 2 years after completion of treatment, final height SDS in cancer survivors can be significantly reduced relative to normative data [57,58]. Short stature, defined as a standing height below the tenth percentile, occurs in approximately 40% of children treated for brain tumors [16]. Cranial irradiation can adversely affect height in childhood cancer survivors [57,59], with reductions in height directly associated with dose and inversely associated with age at exposure [16,60,61]. Reduced stature is also associated with spinal irradiation [62,63,64], intensive chemotherapy-only regimens [52,55,65] and hematopoietic stem cell transplant (HSCT) [66,67]. Adult height Z-scores are lower among those patients who receive both cranial radiation and total body irradiation when compared to those survivors who received total body irradiation or cranial radiation alone [41]. Finally, patients who receive spinal irradiation are at increased risk of skeletal disproportion, with short torsos relative to limb length [68].

It is possible that reduced adult stature could negatively affect self-esteem, social relationships, and functioning among childhood cancer survivors [69]. In a study of 183 young survivors of childhood cancer, Mulhern, *et al.* [70] reported a 2.3-fold (95% CI: 1.0–5.0) higher risk of school-related problems among those with physical disabilities than those without physical disabilities (including short stature), but found no association between physical disability and the activities component of the Child Behavior Checklist [70].

## 3. Musculoskeletal Defects

### 3.1. Bone Density

Deficits in bone mineral density (BMD) occur in children with newly diagnosed ALL [71,72]. Among long-term cancer survivors of various diagnoses, estimates of BMD deficits (defined as lumbar or total body Z-scores below −1) range from 22%–68% [73,74,75,76,77,78,79]. Low BMD is of concern in children treated for cancer, as low BMD is associated with an increased risk of fracture in the general population. Halton, *et al.* reported that approximately 16% of 186 children screened by radiography within 30 days of a diagnosis of ALL had one or more vertebral compression fractures [80]. Moreover, the rate of fracture is 6.1-fold higher than expected in children diagnosed with cancer relative to healthy controls up to one year after completing therapy (95% CI: 3.0–11.3) [72]. Deficits in BMD and subsequent fractures may occur after exposure to methotrexate or corticosteroids [73,77,81,82,83,84,85], particularly prolonged exposure to corticosteroids as a result of graft-*versus*-host disease [86], or because of treatment-related endocrinopathies [87,88,89,90,91]. BMD deficits among children and adolescents with cancer may be potentiated by inadequate calcium, Vitamin D [92] and protein intake, or by higher daily television/computer screen time and low physical activity [90,93,94]. Cancer treatment during adolescence is associated with an increased risk of low BMD in ALL survivors [85,89,95,96] and is likely due to disruption of the normal acceleration in bone mineral accretion occurring during puberty [85].

The long-term implications of low BMD on the risk of fracture and future risk of osteoporosis in childhood cancer survivors are largely unknown [97,98]. However, studies of non-cancer populations show that reductions in BMD during middle and late adulthood increases the risk of fracture [99,100,101], with associated mobility deficits and poor physical function [102,103,104]. The Children’s Oncology Group (COG) Long-Term Follow-Up Guidelines recommend screening for bone density deficits among cancer survivors who have received agents known to adversely affect bone health [6]. Currently, treatments for BMD deficits among childhood cancer survivors include dietary counseling and supplementation to ensure adequate calcium and Vitamin D intake [6].

### 3.2. Deformity

Various skeletal deformities occur in childhood cancer survivors, including axial misalignment of the spine [105,106], limb-length discrepancy [107,108,109], and chest wall abnormalities [110,111]. In many instances, skeletal deformity is the result of irradiation, which can arrest chrondrogenesis in the tubular bones and vertebrae and affect membranous ossification within the flat bones (e.g., skull, pelvis, or ribs), leading to hypoplasia and asymmetric growth [112,113,114]. The most commonly reported skeletal effects of radiotherapy are spinal deformities, such as scoliosis, kyphosis, and lordosis, which are most frequently observed in among survivors of neuroblastoma, medulloblastoma, and Wilms tumor who received abdominal or craniospinal irradiation [115]. The severity of axial misalignment is associated with irradiation before 6 years of age, higher doses of irradiation, larger radiation fields, asymmetric [105,116,117,118] or orthovoltage-based radiation [119], and spinal exposure during the adolescent growth spurt [120]. Spinal deformities are also frequent among survivors of intraspinal tumors [121,122,123,124,125]. Scoliosis that involves thoracic vertebrae contributes to restrictive lung defects, characterized by decreased total lung capacity, reduced tidal volume, and elevated respiratory rate [126,127]. These conditions increase the energy required to breathe, and over time result in respiratory muscle fatigue, respiratory failure, and pulmonary hypertension [127]. Kyphosis and chest wall abnormalities also affect breathing by compressing the lungs, and, in severe cases, cause weakness in the lower extremities by increasing pressure on nerve roots of the spinal cord [128]. Vertebral deformities may interfere with leisure-time physical activities because of their impact on reduced respiratory capacity and physical functioning [129,130].

Craniofacial bony deformities are frequently observed among survivors of retinoblastoma, but are also reported among survivors of head and neck tumors [131,132,133]. Among retinoblastoma survivors, craniofacial deformities typically appear as a cluster of conditions including hypotelorism, enophthalmos, depressed temporal bones, depressed nasion and narrow and deep orbits [134]. Similar late effects, such as enophthalamus and lacrimal duct stenosis are also observed among individuals treated for orbital rhabdomyosarcomas [135,136]. Among patients with sarcomas located on or near the jaw or neck, mandibular muscle and temporamandibular joint function can be compromised [137]. Facial and nuchal asymmetry is common [138,139]. The risk of craniofacial deformities is associated with younger age at diagnosis. Moreover, the craniofacial bones appear to be extremely sensitive to the adverse effects of radiation with abnormalities reported at doses as low as 400 rads [132]. Survivors of childhood cancer report significantly higher rates of scarring and disfigurement compared with siblings for the head and neck (25.1% *vs.* 8.4%) [140].

Limb-length discrepancy is common among Ewing sarcoma survivors treated with irradiation to the extremities [105,107,109,141,142,143] and among Wilms tumor survivors treated with abdominal irradiation [108,119,144]. The biomechanical adaptions necessary to accommodate leg-length differences result in back, flank, hip, and knee pain, arthritis of the hip or knee, psoasitis, iliotibial band syndrome, and uneven gait [145,146,147]. Small differences in limb length can be accommodated by shoe inserts, but differences of more than 4 cm may require surgical intervention. Survivors of childhood cancer are 13 times (95% CI: 6.2–27.3) more likely than their siblings to undergo a leg-lengthening, leg-shortening, or joint replacement surgery [148]. Limb-lengthening and limb-shortening surgeries are associated with a risk of infection, nonunion or malunion of the affected bone, joint dislocation, nerve injury, and fractures after removal of the lengthening apparatus [149,150]. Substantial decreases in strength, power, and function occur immediately after surgery; small residual deficits in muscle strength are likely to persist [151,152,153]. Physical therapy for children and adolescents undergoing leg-lengthening and leg-shortening procedures is required to strengthen muscle and maintain joint flexibility to ensure maximum recovery of function.

### 3.3. Limb Sparing and Amputation

Children with extremity tumors may undergo limb-sparing surgery or amputation for local control. Both procedures are associated with the risk of immediate and long-term loss of physical function, although many children adapt well to limb dysfunction or loss [154,155]. However, a recent report from the Childhood Cancer Survivor Study (CCSS) that included 1094 survivors of extremity tumors (median age 33 years) found that tumor location in the lower extremity, female sex, older age, osteosarcoma tumor type, above-knee amputation, and abdominal irradiation were risk factors for activity limitations [156]. Survivors with upper-extremity tumors were less likely than those with lower-extremity tumors to graduate from college, and non-white children were more likely than white children to experience functional loss, not graduate from college, and be unemployed [156]. Early rehabilitation to restore function, teach the child compensatory strategies, or provide environmental adaptations to maximize function is important for these children.

### 3.4. Osteonecrosis

Osteonecrosis is a disease that results from temporary or permanent interruption of blood supply to the bone. Symptomatic osteonecrosis is characterized by pain, decreased mobility, and microfractures [157,158]. If the site of the lesion involves a joint, range of motion is often compromised, and articular collapse may occur necessitating the need for joint replacement surgery [159]. Osteonecrosis is most common in the knees, hips, and shoulders [160], and typically presents during treatment [161]. Symptomatic osteonecrosis occurs in 1%–9% of children treated for ALL [85,162,163,164,165] and 4%–44% of children receiving hematopoietic stem cell transplant (HSCT) [166,167,168,169]. In contrast, asymptomatic osteonecrosis, detected by magnetic resonance imaging, occurs in 15%–72% of children with ALL [165,170,171,172,173,174]. Although rare, cases of osteonecrosis have been observed among children treated for solid tumors [175]. Risk factors for osteonecrosis include glucocorticoid therapy [161,166,176], irradiation, and older age at diagnosis [162,177]. High BMI [165,167,169] and female sex [85,162,163,164] are also associated with an increased risk of osteonecrosis, but findings are not consistent across studies.

Surgical procedures such as core decompression, arthrodesis, or joint replacement are required in 24%–42% of children with symptomatic osteonecrosis [162,164,178]. Although these procedures may alleviate symptoms in the immediate term, there are concerns about the long-term implications of these procedures for young individuals whose skeletons are still growing and maturing [179]. Osteonecrosis can result in significant deficits in physical function and performance in childhood cancer survivors, which can persist decades after the completion of therapy. In a CCSS study of survivors followed for more than 15 years from diagnosis, 41% of survivors with osteonecrosis reported pain in affected joints at rest and 57% reported difficulties performing daily activities of living, such as climbing stairs, rising from a chair, and opening containers [177]. Moreover, in a study of 943 French survivors of ALL, of whom 2.5% developed osteonecrosis, mean physical composite score of Quality of Life (assessed using the SF-36 questionnaire) was found to be lower among those patients with osteonecrosis when compared to those without [180].

### 3.5. Muscle Weakness

Developing skeletal muscles of children are extremely sensitive to the adverse effects of ionizing radiation. Exposure to ionizing radiation during childhood can lead to multiple abnormalities including hypoplasia, atrophy and fibrosis, which in turn can adversely affect lean-body mass, muscle strength, and performance as well as increase the risk of deformity. The nature of the late effect is highly dependent on the radiotherapy site. For instance, survivors of abdominal flank tumors, such as Wilms tumor and neuroblastoma, may develop flank or paraspinal muscular atrophy after receiving 15–45 Gray of abdominal irradiation [106,117,181]. Hypoplasia and tissue fibrosis are among the most common musculoskeletal late effects observed among survivors of soft tissue sarcoma located within the abdomen or pelvis [182,183]. Similarly, muscular atrophy affecting the leg and face occurs in survivors treated for extremity Ewing sarcoma [141] and head and neck tumors [114], respectively.Radiation-induced fibrosis among survivors of head and neck tumors can cause symptoms such trismus, cervical dystonia and facial lymphedema. Muscular atrophy and fibrosis are dependent on radiation dose [141] and may lead to deformity after asymmetric irradiation [184].

Deficits in muscular strength and endurance among survivors of childhood cancer are associated with exposure to specific chemotherapeutic agents in childhood [185,186]. Among 43 female survivors of ALL evaluated at an average of 8 years, deficits in muscle endurance (measured by situp and pushup tests) were seen in survivors treated with asparaginase compared with age-matched controls [187]. Impaired knee extension strength, walking efficiency, and flexibility was reported in 15 ALL survivors (median age 29.9 years) treated with intrathecal methotrexate at a cumulative dose of ≥215 mg/m^2^ compared with those who did not receive methotrexate [188]. Furthermore, children and adult survivors receiving vincristine have reduced peripheral muscle strength (dorsiflexors of ankles and wrists and pinch grip) [189] and increased risk of restricted range of motion [188], respectively. Compared to siblings, childhood cancer survivors perform poorly on tests of lower-extremity strength and mobility despite reporting similar levels of physical activity [190]. Poor physical performance is not always attributable to subclinical cardiac dysfunction [191].

Low lean body mass and reduced muscle strength are associated with high rates of morbidity among childhood cancer survivors. In the St. Jude Lifetime Cohort Study, frailty (defined as presence of ≥3 of the following: low muscle mass, weakness, self-reported exhaustion, low energy expenditure, and slow walking speed) [192] was associated with a 2.2-fold (95% CI: 1.2–4.2) increased risk of developing a new-onset severe or life-threatening chronic condition (grade 3 or 4, Common Terminology Criteria for Adverse Events) [193]. Moreover, the risk of death was 2.6 times higher greater among survivors who were frail than those who were not (95% CI: 1.2–62).

## 4. Neurosensory Deficits

### 4.1. Vision

Visual impairments are reported among children receiving cancer therapy and among long-term survivors. According to a CCSS report [194], long-term survivors (*n* = 14,362) were at higher risk for legal blindness (Relative Risk [RR]: 2.6; 95% CI: 1.7–4.0), cataracts (RR: 10.8; 95% CI: 6.2–18.9), double vision (RR: 4.1; 95% CI: 2.7–6.1), dry eyes (RR: 1.9; 95% CI: 1.6–2.4), and glaucoma (RR: 2.5; 95% CI: 1.1–5.7) than their siblings (*n* = 3901). The median times from cancer diagnosis to the self-reported onset of legal blindness, cataracts, double vision, and dry eyes was 1 year, 1.7 years, 2.2 years, and 7.2 years, respectively [194]. Among childhood cancer survivors with mixed diagnoses, the prevalence of visual impairments was 5.7% [194] and 11.6% [195] at a minimum of 5 and 9 years from diagnosis, respectively. In children who underwent HSCT, the prevalence of cataract was 21% at 5 years, 32% at 10 years, and 36% at 15 years [196]. In a study of 27 survivors treated for retinoblastoma, 26 developed visual fields deficits after a mean follow-up of 21.8 years [197]. Radiation complications among 141 long-term survivors of retinoblastoma at the 5-year follow-up included non-proliferative maculopathy (25%), papillopathy (26%), cataract (31%), and glaucoma (11%) [198]. Ocular deficits are also common among survivors of CNS tumors [199,200]. Radiotherapy involving the eye is a risk factor for cataracts, legal blindness, dry eyes, and double vision [176,177,181,201]. Treatment with prednisone is associated with an increased risk of cataracts [194], whereas treatment with vincristine [202,203,204], cytarabine [205], or doxorubicin [206], is associated with an increased risk of optic neuropathy, keratoconjunctivitis, and conjunctivitis, respectively. Visual impairments can cause significant impairment in physical functioning, performance limitations, and disability [207,208,209,210]. Among cancer survivors, visual impairments have been associated with low physical activity levels with as little as 25% of survivors reaching recommended levels for physical activity [211].

### 4.2. Vestibular Function and Balance

Children with cancer are at risk for disordered balance [212,213,214]. Treatment with platinum compounds can damage the organs of the inner ear and impair vestibular function, resulting in delays in the development of motor skills and recurrent episodes of dizziness and vertigo [215]. Exposure to vinca alkaloids increases the risk of sensorimotor neuropathies and compromised proprioceptive feedback from muscles and joints [216]. Assessments of gross motor performance among children receiving therapy for ALL [212] and among young survivors indicate poor performance on balance sub-scales relative to controls [217,218]. Adult survivors of childhood-onset CNS tumors also have impaired balance compared with normal controls [210,214], occurring as a result of the late effects of tumor infiltration and removal and exposure to cranial radiotherapy [219,220]. Although few studies have evaluated the effects of balance deficits on function among survivors of childhood cancer, findings from the aging literature indicate that balance deficits can adversely affect functional performance [221] and increase the risk of falls [222,223] and injury [224].

### 4.3. Peripheral Neuropathy

Peripheral sensory and motor nerves, which relay information to and from the central nervous system (CNS) and musculoskeletal system, can be adversely affected by cancer therapies [216]. Clinical symptoms include painful dysesthesia, loss of vibration, temperature, and proprioception sensation [225,226], and loss of motor function, and are associated with exposure to platinum agents [227] or vinca alkaloids [228,229,230]. Platinum-induced neuropathies are typically sensory in nature. Symptoms worsen for several months or years after cessation of treatment and new symptoms may appear weeks after the final dose, a phenomenon known as “coasting” [225,231]. In contrast, vinca alkaloids affect both sensory and motor neurons, resulting in a mixed sensorimotor neuropathy that includes motor symptoms such as weakness of the extensor muscles of the fingers, wrists, toes, and of dorsiflexors of the ankle; muscle cramps; and postural hypotension [228,229,230]. Drug interaction between vincristine and antifungal azoles may exacerbate neuropathy [232]. Although peripheral neuropathies are often dismissed as transient side effects of chemotherapy, many survivors experience long-term neuropathy [186,229].

## 5. Neuromotor Deficits

The etiology of neurological motor deficits in children treated for cancer is multifactorial. Neuromotor deficits result from damage to regions of the CNS that control movement or from damage to peripheral nerves [233]. Among children with brain tumors, a space-occupying lesion, compression on adjacent structures, and increased intracranial pressure can produce neurological symptoms. In some instances, surgical resection of a tumor may worsen already-existing neurological problems or lead to the development of new symptoms [234,235]. Radiotherapy-induced neurological deficits are attributed to parenchymal necrosis or infarction in the radiation field [236]. Exposure to vincristine is associated with peripheral motor neuropathy. Neurological late effects are most common in survivors of brain tumors, but also occur in children with ALL, rhabdomyosarcoma of the head and neck with meningeal extensions [237], or neuroblastoma [238].

Ataxia, which is the inability to voluntarily coordinate movement, is possible in children with brain lesions that involve the cerebellum. Ataxia is common among children with posterior fossa lesions and can manifest as dysarthria (difficulty with speech), dysphagia (problems in swallowing), dysmetria (difficulty in reaching for objects), gait changes, tremors, and hypotonia (low muscle tone) [239]. Among children with brain tumors, the occurrence of neurological deficits is highest immediately after following surgical resection of the tumor [235,240,241]. Although the overall number of neurological deficits may decrease and the severity of symptoms may lessen with time [235,241], mild symptoms such as impaired eye abduction, dysmetria, and dysarthria can persist for more than 5 years after treatment for a childhood brain tumor [240,242,243]. In a large cross-sectional report from the CCSS, 49% and 26% of survivors of childhood brain tumors reported coordination or motor problems, respectively [244]. Younger age at treatment [245,246,247], gross tumor volume [236], hydrocephalus [243], and a history of shunt placement [236] are associated with increased risk of neurologic morbidity.

Cerebellar mutism syndrome, or posterior fossa syndrome, is a serious neurological condition that can occur following surgery involving the posterior fossa [248,249,250]. This syndrome is characterized by delayed-onset mutism followed by dysarthria, profound ataxia, and cranial nerve palsies. Symptoms typically appear within 1–2 days of surgery and may take many days to months to resolve. Cerebellar mutism syndrome is reported in 20%–24% of children with medulloblastoma or primitive neuroectodermal tumors [251,252]. Mild neurological dysfunction is permanent for many patients with this syndrome [251].

Facial and limb paresis, hemiparesis, and paralysis also occur in children receiving cancer therapy [243,253]. Depending on the site affected, symptoms can include difficulty holding or grasping items or problems with balance and gait. In turn, severe imbalances in muscle activity and reduced mobility can lead to additional complications such as muscle and joint stiffness, muscle spasms, contracture, and loss of aerobic fitness. Facial and limb paresis are reported among patients 5 years after surgery for a brain tumor [240]. In addition, mild to severe paresis and paraplegia are reported among survivors of neuroblastoma, occurring as a result of intraspinal tumors, or complications following surgery [238].

Deficits in gross and fine motor skills occur in childhood cancer survivors, particularly those treated for ALL, and are often attributed to damage to the peripheral nervous system. Impairments in ankle dorsiflexion [254] and strength [255] are reported in children receiving ALL therapy. Disturbances in gross motor skills can be significant because are required for normal development of walking, running, and jumping [256]. Among survivors, ankle dorsiflexors and passive dorsiflexion [189], and knee strength [185,187] may also be impaired. In a study of 75 individuals who survived an average of 24.6 years from diagnosis of ALL, Ness, *et al.* reported that lower-leg strength was associated with poorer function as measured by the Timed Up and Go and 2-min walk tests [17]. Early identification of children at risk for gross motor neuropathies is important to initiate interventions, such as individualized stretching and strengthening programs and bracing, to ameliorate physical impairments, and to minimize the risk of long-term functional loss [257]. Peripheral neuropathies can also adversely affect the fine motor skills needed for learning [258] and the ability to manipulate objects by using the thumb and fingers. Difficulties with handwriting (measured by drawing speed, pause duration, and pen pressure) are more frequent in children treated for ALL than in age-matched healthy controls [259]. Although deficits in fine motor skills can occur after completion of treatment [260], these findings have not been confirmed in all studies [261,262].

A rare but serious complication following mantle radiation among survivors of Hodgkin lymphoma is severe atrophy and weakness of the cervical and shoulder girdle musculature [263], particularly the neck extensors. As a result, individuals adopt a posture in which the head is flexed forward, often with the chin resting on the chest. Individuals are unable to extend the neck thus lifting the chin, leading to a condition referred to as Dropped-head Syndrome [264]. Weakness can manifest more than 10 years following radiotherapy exposure with symptoms progressively worsening over time. Weakness of the neck flexors is also common among Hodgkin lymphoma survivors exposed the mantle radiation [265]. Dropped-head Syndrome is a disabling condition interfering with daily activities such as driving a car, working on a computer, reading and lifting heavy items [265]. Attempts to treat this disorder through surgery and physical therapy have been largely unsuccessful [266].

Neurological motor deficits that impair physical function can influence daily activities of living and affect an individual’s ability to participate in expected roles at home, school and work. A study by Ness, *et al.* reported that 155 brain tumor survivors (median age of 22 years [range, 18–58] at evaluation) had lower grip strength, knee extension strength, and exercise tolerance (peak oxygen uptake) than healthy controls [210]. Moreover, survivors with physical performance limitations (measured by the Physical Performance Test) were more likely to reside as dependents (95% CI: 2.0–12.2) and to not attend college than were controls (OR: 2.3, 95% CI: 1.2–4.4) [210]. Brinkman, *et al.* reported that brain tumor survivors (*n* = 78) were more likely than healthy adults to avoid aspects of their physical environment (e.g., traversing a single flight of stairs, uneven terrain, and traveling to unfamiliar places) and less likely to engage in social activities (e.g., going to a bank, restaurant, or friend’s home) [267]. Interestingly, survivors with poor physical performance scores were more likely than survivors without performance deficits to report restricted environmental access [267]. Although neurological late effects are most frequently observed in survivors of brain tumors, they can also affect physical performance in survivors of other cancers. For instance, neurological deficits occur in up to 30% of survivors of neuroblastoma. Neuroblastoma survivors with physical performance limitations have lower rates of high-school graduation, higher rates of unemployment, and lower annual household incomes than do survivors without physical performance limitations [268].

## 6. Cardiopulmonary Impairments

Childhood cancer survivors are at increased risk of cardiac and pulmonary toxicities during and after therapy. Symptomatic cardiac dysfunction occurs in 1%–2% of children during anthracycline chemotherapy [269]. Acute cardiotoxicity is also reported among children treated with high doses of cyclophosphamide prior to HSCT [270,271], and among those whose treatment regimen includes chest radiation [272,273]. Clinically detectable heart failure is rare during treatment; acute changes usually manifest as either transient depression of myocardial contractility [274,275] or nonspecific ST-segment and T-wave abnormalities [276]. Acute respiratory toxicity is also possible during treatment (rates range from 3%–17%) [277,278,279] and is most common among children exposed to whole lung or chest directed radiation [277], and those treated with bleomycin, busulfan or nitrosoureas as part of chemotherapy or transplant preparatory regimens [278,280,281]. HSCT recipients who develop graft *versus* host disease are at increased risk for bronchiolitis obliterans, a condition characterized by interluminal and peribronchial fibrosis with potential to completely obstruct small terminal airways [282,283,284,285]. In adult trials, newer therapies/targeted agents are also implicated in acute pulmonary toxicity, including gemcitabine [286,287], tyrosine kinase inhibitors [288,289], monoclonal antibodies [290,291], and mammalian target of rapamycin inhibitors [292,293]. Acute lung injury typically manifests as pneumonitis and is exacerbated when chemotherapeutic agents with known toxicities are given concomitantly with chest or lung-directed radiation [294].

Children who survive these acute toxicities can recover, but early toxicity can be associated with chronic disease as these children age. For example, Lipshultz, *et al.* reported acute cardiac toxicity (within one year of treatment) among 11 of 115 patients treated with doxorubicin for ALL, all of whom were successfully treated with medication. Although medications were eventually discontinued in all cases, five of the 11 patients subsequently developed congestive heart failure 3.7–10.3 years after doxorubicin exposure [295]. Motosue, *et al.* examined pulmonary function values in 10 children with solid tumors before and after exposure to whole lung radiation and reported an immediate decline in forced vital capacity, forced expiratory volume in 1 second, total lung capacity, and diffusing capacity of the lung for carbon monoxide. Subsequent measures indicated three years of improvement, followed by a gradual decline in lung function [296]. Children with acute toxicities should be treated with appropriate medications and rehabilitative services if needed for optimal recovery; once recovery is optimized, monitoring according to clinical practice guidelines developed by cooperative group, such as the COG Long-Term Follow-Up Guidelines [6], is recommended (Table 2).

Childhood cancer survivors are at increased risk for both long-term cardiac and pulmonary impairments, often many years after therapy. Cardiac late effects include valve dysfunction, coronary artery disease, arrhythmias, and cardiomyopathy. Cardiac problems are typically asymptomatic soon after completion of treatment, with evidence of clinical disease typically emerging years later. Using data from the Childhood Cancer Survivor Study (CCSS), Mulrooney, *et al.* reported cumulative incidences of 4.1%, (95% CI: 3.2%–5.0%) for congestive heart failure, 4.0% (95% CI: 3.1%–4.9%) for valvular abnormalities, and 3.0% (95% CI: 2.1%–3.9%) for pericardial disease 30 years after diagnosis [297]. Furthermore, rates of any cardiac adverse event were 5–6-fold higher among survivors when compared to siblings [297]. Hudson, *et al.* report higher rates in the St. Jude Lifetime Cohort, where survivors are screened to identify disease. The overall prevalence was 4.5% (95% CI: 3.5%–5.5%) for cardiomyopathy, 29.2% (95% CI: 27.1%–31.5%) for valvular disease, and 7.4% (95% CI: 6.2–8.7) for arrhythmia a median of 25 years (interquartile range 20–31) post treatment [3]. Research related to cardiac late effects among cancer survivors now includes a focus on the identification of imaging technologies and biomarkers of late onset cardiac problems so that disease might be detected and treated sooner to prevent early cardiac death in this vulnerable population. Importantly, recent data suggests that survivors who adopt a healthy lifestyle and who do not develop traditional predictors of cardiovascular disease, that is, hypertension, obesity, dyslipidemia, and insulin resistance, have decreased risk for adverse cardiac outcomes [298]. Additionally, exercise intervention in those with reduced cardiac function appears to be safe and may also be efficacious [299].

Long-term pulmonary problems among survivors of childhood cancer include fibrosis, recurrent pneumonia, chronic cough, pleurisy, need for supplemental oxygen, abnormal chest wall, exercise induced shortness of breath, bronchitis, and chronic obstructive pulmonary disease [300]. Mertens, *et al.*, using data from the CCSS, reported rates per 1000 son-years of 0.9 (95% CI: 0.7–1.1) for lung fibrosis, 4.2 (95% CI: 3.8–4.5) for need for supplemental oxygen, 4.0 (95% CI: 3.7–4.4) for chronic cough, 6.7 (95% CI: 6.2–7.2) for exercise induced shortness of breath, and 13.7 (95% CI: 12.9–14.4) for bronchitis with onset at least five years after diagnosis. The relative risks, comparing survivors to siblings, for an adverse pulmonary outcome ranged from 1.4 (95% CI: 1.1–1.8) for pleurisy to 8.6 (95% CI: 3.9–18.8) for an abnormal chest wall [301]. In the St. Jude Lifetime Cohort, pulmonary function abnormalities were prevalent in 65.2% of childhood cancer survivors exposed to busulfan, carmustine, lomustine, bleomycin, thoracic radiation or thoracotomy, screened a median of 25 years post diagnosis [3]. Prevention of pulmonary toxicities is focused on reducing exposure to radiation and known pulmonary toxic chemotherapies to minimize acute pneumonitis. For long-term survivors, preventing infection and further damage is vital in addition to screening. Influenza and streptococcal pneumonia vaccines are recommended for childhood cancer survivors, and they are advised not to smoke [294].

Prevention and remediation of adverse cardiac and pulmonary health among childhood cancer survivors is extremely important. Death from cardiac and pulmonary causes among childhood cancer survivors are exceeded only by death from second malignant neoplasms with excess risks of 7.0 (95% CI: 5.9–8.2) and 8.8 (95% CI: 6.8–11.2) when compared to age- and sex-matched members of the general population [302]. Adequate cardiac and pulmonary function is essential for fitness and mobility and when impaired has a profound impact on physical health [303,304], cognition [305], and on participation in daily life [210,306]. Adult survivors of childhood cancer are less active than their siblings [307]; those with chronic health conditions continue to demonstrate declining levels of activity as they age [49]. Poor fitness and inadequate levels of physical activity appear to start early in survivorship [190], perhaps indicating a need for rehabilitation programs [308] tailored to meet the needs of childhood cancer survivors whose treatment-related late effects may subtly or apparently interfere with abilities to be active in sport or leisure activities that require endurance and coordinated movement [309].

**Table 2:** Screening recommendations for cardiopulmonary late effects according to the Children’s Oncology Group Long-Term Follow-Up Guidelines.

Condition	Screening Recommendations
Arrhythmia, cardiomyopathy	Fasting glucose and lipid profile every 2 years, refer as indicated. ECHO and/or MUGA at baseline, then periodically as indicated. EKG at baseline.
Left ventricular dysfunction	ECHO or MUGA at baseline, then periodically as indicated. EKG at baseline, repeat as indicated.
Atherosclerotic heart disease, congestive heart failure, myocardial infarction, pericardial fibrosis, pericarditis, valvular disease	Fasting glucose and lipid profile every 2 years, refer as indicated. ECHO at baseline, then periodically as indicated. EKG at baseline, repeat as indicated.
Hypertension	Yearly physical, blood pressure, screening blood work at baseline, repeat as clinically indicated.
Acute respiratory distress, bronchial obliterans, bronchiectasis, chronic bronchitis, fibrosis, interstitial pneumonitis, pulmonary dysfunction, obstructive lung disease, restrictive lung disease	Chest X-ray. PFTs (including DLCO and spirometry) at baseline, repeat as clinically indicated.
Chronic sinusitis	Annual history and physical, nasal/sinuses exam.

**Abbreviations:** ECHO, echocardiogram; EKG, electrocardiogram; MUGA, multi-gated acquisition scan; PFT, pulmonary function test; DLCO, diffusing capacity of the lungs for carbon monoxide.

## 7. Conclusions

Individuals successfully treated for cancer are often faced with a unique set of health problems as they age because of the therapies received in childhood. These chronic health problems may limit physical performance, and, in turn, interfere with functional capacity and participation in work, social, and recreational activities. Nevertheless, limited studies exist describing the impact of many chronic conditions, such as obesity, short stature, peripheral neuropathy, poor balance, and neuromotor impairments, on function. It is also unclear how poor mobility and function may contribute to the etiology of new chronic conditions above that of treatment-induced organ toxicity. For instance, poor mobility could reduce participation in physical activity and recreational pursuits such that overtime, cardiovascular health is adversely affected, or reduced muscle function could promote early-onset frailty. Accordingly, additional research characterizing the etiology of functional impairments among cancer survivors using robust and objective measures, and accurate assessment of risk factors, including medical, psychosocial, lifestyle and treatment-related factors, are required. Moving forward, interventions to prevent, ameliorate, or delay the onset of health conditions among children treated for cancer are likely to become an increasingly important area in survivorship research. In particular, interventions directed at lifestyle behaviors represent key areas of potential research. Interventions and prevention programs trialed in the general population or in survivors of adult-onset cancers that have targeted the chronic diseases for which childhood cancer survivors are at risk, such as low BMD [310] and peripheral neuropathy [311], may be useful in survivors. Also, because many aspects of physical health are interrelated, some interventions may benefit a survivor’s health in multiple ways. For instance, high levels of physical activity and appropriate calorie restriction may have the more immediate effect of reducing obesity. However, good lifestyle choices sustained over the long term could prevent or delay the onset of conditions, such as bone and lean muscle loss, and minimize the risk of future cardiovascular disease. Finally, continued follow-up and screening will remain an important aspect of care for many chronic conditions that affect childhood cancer survivors for which the trajectory over time is unknown.
